# Molecular Signatures of Quiescent, Mobilized and Leukemia-Initiating Hematopoietic Stem Cells

**DOI:** 10.1371/journal.pone.0008785

**Published:** 2010-01-20

**Authors:** E. Camilla Forsberg, Emmanuelle Passegué, Susan S. Prohaska, Amy J. Wagers, Martina Koeva, Joshua M. Stuart, Irving L. Weissman

**Affiliations:** 1 Institute for Biology of Stem Cells, Department of Biomolecular Engineering, University of California Santa Cruz, Santa Cruz, California, United States of America; 2 The Eli and Edythe Broad Center for Regeneration Medicine and Stem Cell Research, University of California San Francisco, San Francisco, California, United States of America; 3 Institute of Stem Cell Biology and Regenerative Medicine, Departments of Pathology and Developmental Biology, Stanford University School of Medicine, Stanford, California, United States of America; 4 Joslin Diabetes Center, Harvard Stem Cell Institute and Department of Stem Cell and Regenerative Biology, Harvard University, Cambridge, Massachusetts, United States of America; Katholieke Universiteit Leuven, Belgium

## Abstract

Hematopoietic stem cells (HSC) are rare, multipotent cells capable of generating all specialized cells of the blood system. Appropriate regulation of HSC quiescence is thought to be crucial to maintain their lifelong function; however, the molecular pathways controlling stem cell quiescence remain poorly characterized. Likewise, the molecular events driving leukemogenesis remain elusive. In this study, we compare the gene expression profiles of steady-state bone marrow HSC to non-self-renewing multipotent progenitors; to HSC treated with mobilizing drugs that expand the HSC pool and induce egress from the marrow; and to leukemic HSC in a mouse model of chronic myelogenous leukemia. By intersecting the resulting lists of differentially regulated genes we identify a subset of molecules that are downregulated in all three circumstances, and thus may be particularly important for the maintenance and function of normal, quiescent HSC. These results identify potential key regulators of HSC and give insights into the clinically important processes of HSC mobilization for transplantation and leukemic development from cancer stem cells.

## Introduction

Hematopoietic stem cells (HSC) are rare, multipotent, self-renewing precursor cells capable of generating each and every specialized cell of the blood system. Precise regulation of HSC proliferation and cell fate decisions is necessary to maintain ongoing production of mature blood cells throughout adult life and for rapid, regenerative responses to hematologic injury. Several lines of evidence indicate the importance of active maintenance of HSC stem cell function. The regulation of HSC quiescence in the bone marrow (BM) niche is of particular importance [1], [2]. Several recently identified genes that perturb HSC quiescence also disrupt stem cell maintenance and homeostatic blood cell production. Many of these encode transcription factors or cell cycle regulators that directly modulate the proliferative activity of HSC. Others encode soluble mediators, produced by niche cells that act extrinsically to activate HSC proliferation. Together, these data suggest that precise control of cell division is crucial for appropriate stem cell behavior and that the proliferative activity of HSC is normally restricted by both HSC intrinsic factors and extrinsic factors produced in the HSC niche. Elucidating the molecular pathways that maintain HSC quiescence will thus enable directed manipulation of HSC function endogenously and in the context of hematopoietic cell transplantation.

Although the tightly regulated equilibrium between HSC quiescence and proliferation is clearly important for long-term maintenance of stem cell function, some physiologic functions of HSC require short-term perturbation of this balance. For example, during stem cell differentiation, which is required for normal blood cell production, HSC exit quiescence to generate more mature multi- and oligopotent progenitor cells, with concurrent loss of self-renewal potential and gain in proliferative activity [3]. In addition, stem cells can be “mobilized” in response to hematopoietic stress, rapidly undergoing expansion and migration to repopulate the peripheral hematopoietic compartments. Stem cell function is also perturbed during leukemogenesis, in which oncogenic transformation derails the normal state of HSC to promote proliferation, metastasis to extramedullary sites, and the production of abnormal blast cells. Significantly, while differentiation of HSC to multipotent progenitors (MPP) is associated with increased proliferation and loss of self-renewal activity [4]–[6], mobilization and transformation of HSC induces proliferation without loss of self-renewal potential, demonstrating that a highly proliferative state does not preclude maintenance of long-term self-renewal. A complete understanding of the complex regulatory networks that govern HSC function remains an essential, but elusive, goal.

Elucidation of stem cell regulatory mechanisms in many systems has been advanced greatly in recent years by the application of genome-wide profiling approaches to characterize gene regulatory networks in purified stem cell populations. To specifically investigate the mechanisms by which normal HSC are maintained, we have examined how the expression profile of normal, quiescent HSC changes under three different scenarios: normal differentiation into multipotent progenitors (MPP); cytokine-induced expansion and mobilization; and leukemic transformation. When considered individually, these gene expression profiles provide significant molecular insights to the regulation of each of these specific processes. Moreover, when considered collectively, these data identify alterations in gene expression that are common to all three conditions assayed. This meta-analysis defines a signature profile of normal, quiescent stem cells present in the BM of mice and reveals novel molecular pathways that are commonly altered when HSC are uprooted from their normal quiescent niche. Given that HSC are the essential functional units in BM transplantation, the identification of factors that regulate their maintenance and function will likely improve treatments for hematodeficiency and hematopoietic cancers. Thus, these studies provide insights into the normal differentiation and mobilization of HSC, and the molecular mechanisms by which these processes are usurped or dysregulated during oncogenesis and cancer metastasis.

## Results

### Cytokine-Induced Mobilization of HSC

In adult mice and humans, the majority of HSC are found in the BM, although HSC are also constitutively present at low levels in the circulation [7], [8]. The frequency of HSC in the blood can be significantly increased through the use of mobilizing agents, including cytotoxic drugs and/or cytokines, which act to both drive HSC proliferation and to induce HSC migration from the BM into the bloodstream. In particular, treatment of mice with a combination of the chemotoxin cyclophosphamide (Cy) - which kills mainly proliferating hematopoietic progenitors and very few of the G_0_ HSC - plus the cytokine granulocyte-colony stimulating factor (G-CSF or G) induces a rapid and reproducible expansion and migration of BM HSC [9]–[11]. Following administration of Cy + 2 daily doses of G, the BM HSC population (referred to hereafter as mobHSC) expands dramatically, reaching ∼10–12 times the size of the HSC compartment in normal animals [9]. Expansion of HSC in this early phase of mobilization occurs only in the BM [11], and appears to be associated with an increase in HSC self-renewal and an accelerated rate of HSC division, as measured by incorporation into newly synthesized DNA of the thymidine analog bromodeoxyuridine (BrdU) [3]. While BM HSC of unmanipulated animals are largely quiescent (<10% of cells in S-G_2_/M phases of the cell cycle), up to 35% of mobHSC exhibit >2n DNA content, and ∼85% of these cells incorporate BrdU after only 12 hours of labeling [3]. After day +2, HSC frequencies in the BM decline as HSC migrate from the marrow and begin to appear in significant numbers in the blood and spleen of mobilized animals [9], [12].

**Figure 1:**
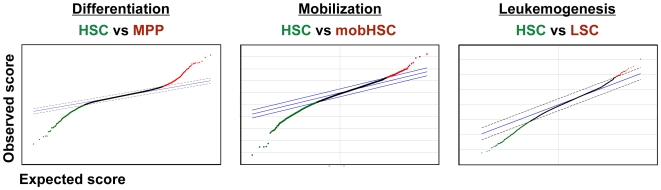
Significance Analysis of Microarray (SAM) plots. Pair-wise comparison of gene expression during HSC differentiation, mobilization, and leukemic transformation. Features colored green or red represent genes up- or downregulated, respectively, in each comparison.

To identify specific molecular mediators of the proliferative and migratory responses of HSC to Cy/G-induced hematopoietic stress, we used DNA microarray technology to identify changes in gene expression associated with HSC mobilization. This analysis yielded a dataset comprised of 2,611 targets exhibiting differential expression (**Figure 1**); 2,461 clones (representing 1,670 individual genes) were significantly downregulated in mobHSC as compared to untreated BM HSC (**Supplemental Table S1**), while only 150 clones (representing 97 individual genes) (**Supplemental Table S2**) were significantly upregulated in response to Cy/G treatment. This bias towards reduced gene expression upon mobilization may suggest that downregulation of genes actively maintaining HSC quiescence is an important mechanism of cytokine-induced HSC expansion.

Functional classification of genes differentially expressed by non-mobilized versus mobHSC revealed that ∼half of these encode unknown genes or uncharacterized EST sequences. Gene Ontology (GO) analysis [13] was performed to functionally classify the known genes, and revealed significant enrichment among up-regulated genes of cell cycle regulators, translation and RNA processing factors, and genes involved in basic cell metabolic processes. These findings are consistent with the robust activation of proliferation and uniform exit from quiescence induced in HSC by Cy/G treatment. Among the significantly down-regulated genes, a sizable proportion encoded transcription factors, signaling proteins, and cell cycle associated proteins, largely inhibitors of cell cycle progression (**Supplemental Table S1**).

### Leukemic Transformation of HSC

Leukemia is an aberrant hematopoietic process that can be initiated and sustained by rare leukemic stem cells (LSC) that drive the formation and growth of tumors [14]–[17]. Recent evidence indicates that LSC can emerge either from transformed HSC [17], [18] or from transformed progenitor cells that have re-acquired the stem cell property of self-renewal [15]. In one model, preleukemic progression of a myeloproliferative disorder occurs in the only self-renewing cells of the myeloid lineage, HSC, but emerges from these clones when self-renewal is not shut down [or re-emerges] when these preleukemic or chronic phase leukemic HSC develop multipotent or oligolineage progenitors at acute or blast crisis phases of the disease [19]. Developing interventions that specifically target LSC is an appealing strategy for improving the specificity and efficiency of cancer treatment [20]. Such targeting will require understanding of how LSC escape normal regulatory mechanisms to become malignant. Myeloid malignancies provide an excellent opportunity for addressing these fundamental questions at the cellular and molecular level as relevant LSC populations have been identified and can be distinguished and isolated apart from other cells in the tumor [21].

Mice lacking the AP-1 transcription factor JunB in hematopoietic cells develop a myeloproliferative disorder (MPD) that accurately reproduces important clinical aspects of human leukemias including chronic myelogenous leukemia (CML) and chronic myelomonocytic leukemia (CMML). We have identified the LSC population in these mice as arising from the HSC compartment [17], [18], [22]. Loss of JunB function in HSC causes an aberrant stem cell expansion leading to MPD development and eventually frank leukemia. Inactivation of *jun*B endows HSC with proliferative and survival advantages, two key properties necessary for HSC transformation into LSC.

To identify molecular mediators of leukemic transformation in *jun*B-deficient HSC, we performed gene expression microarray analysis. Because gene expression profiles of LSC may differ depending on the stage of the disease, we investigated the LSC population of each leukemic mouse separately and selected for analysis only those mice exhibiting advanced MPD but no blast crisis progression. This strategy was designed to uncover genes involved in the initial steps of HSC transformation caused by loss of JunB expression, which in turn mediates the early phase of leukemia development.

RNA was isolated and amplified from 4,000–8,000 double-sorted HSC from the BM of individual *jun*B-deficient mice. Target transcripts significantly up- or down-regulated were identified by Statistical Analysis of Microarrays (SAM) [23] of six independent arrays according to the criteria described previously [24]. These analyses detected 1,203 targets, representing 958 individual genes, that were significantly downregulated in *jun*B-deficient HSC (**Figure 1** **and Supplemental Table S3**). In contrast, only 26 target cDNAs, representing 23 individual genes, were upregulated in the same population (**Supplemental Table S4**). This bias towards reduced gene expression suggests that JunB acts primarily as a transcriptional activator in HSC and suggests that downregulation of genes actively maintaining HSC function is an important prerequisite for their leukemic transformation.

### Meta-Analysis Reveals a Normal HSC Signature

**Figure 2:**
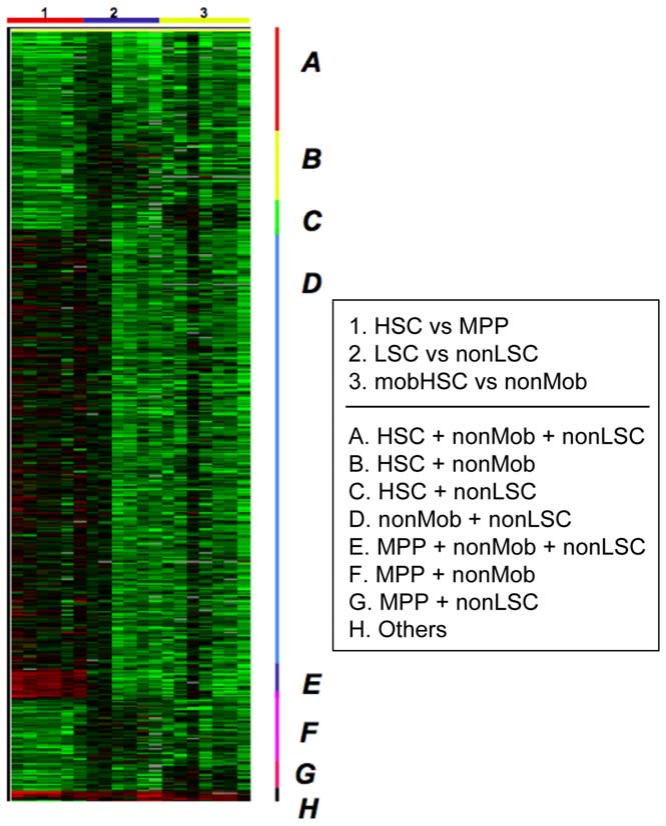
Heatmap representation of differential gene expression. Each of the 18 columns represents a pairwise comparison. Rows represent individual genes. Note that “nonLSC” and “nonMob” are the normal HSC counterparts to LSC and mobilized HSC.

**Figure 3:**
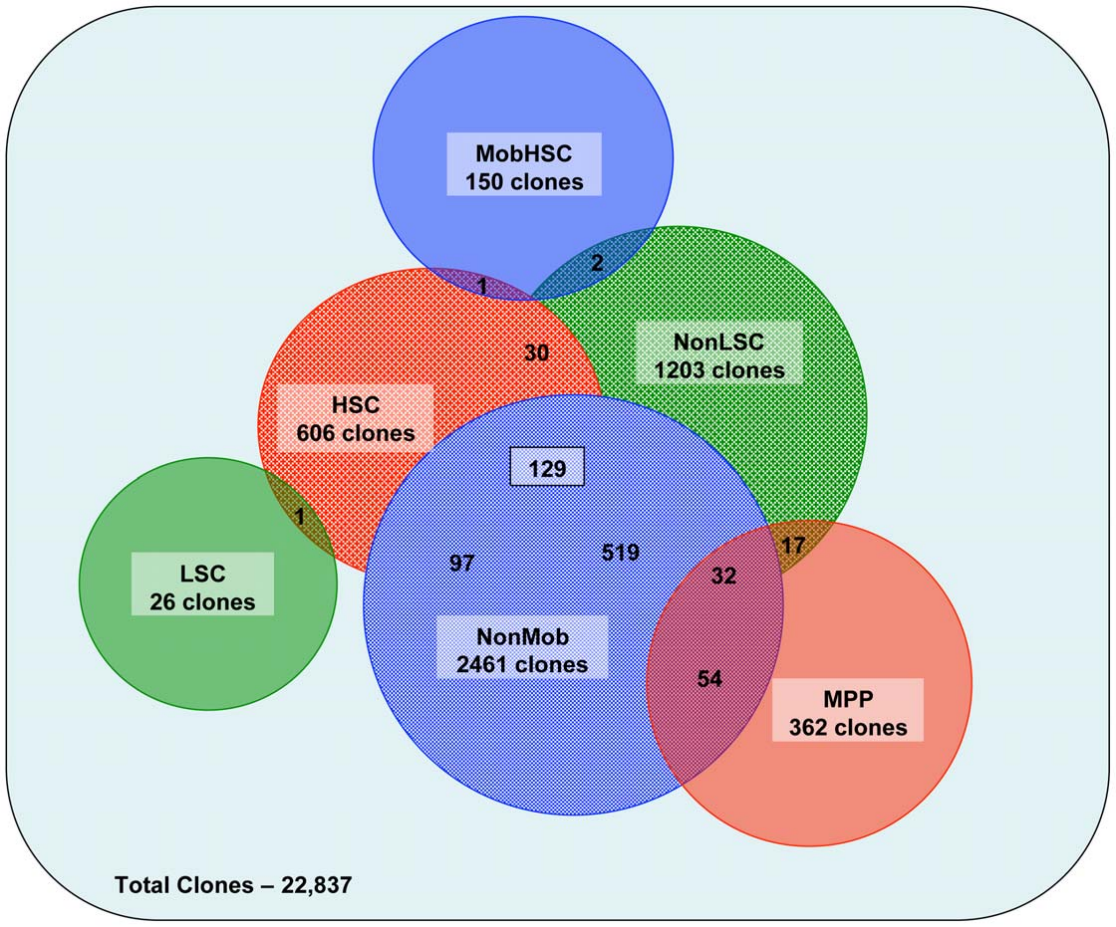
Venn diagram representation of differential gene expression. Lists of differentially expressed clones in each pairwise comparison were compared to identify clones that are shared or unique to different HSC fates. The numbers represent the number of up- or downregulated clones in each pairwise comparison and the number of clones shared between comparisons.

We previously employed gene expression profiling to study changes in gene regulation associated with the differentiation of self-renewing HSC to MPP [24], uncovering many novel pathways that regulate normal HSC function and specify HSC differentiation. Thus, together with the mobilization and leukemogenesis arrays described above, we have performed 3 pairwise comparisons to identify changes of gene expression associated with the differentiation, mobilization, or transformation of purified HSC ([24]; **Figure 1**). A heatmap representation of these comparisons is presented in **Figure 2**. These three distinct processes have in common some characteristic cellular responses, for example the exit of HSC from quiescence and entry of these cells into cycle. To determine whether common molecular pathways mediate such shared HSC responses during differentiation, mobilization, and leukemic transformation, we next performed a meta-analysis of the differentially regulated genes from these three pairwise comparisons. We searched for significant two-way and three-way overlaps among the differentially expressed gene lists identified in the HSC vs. MPP, mobilized (Mob) vs. non-mobilized (nonMob), and leukemic (LSC) vs. non-leukemic (nonLSC) HSC datasets (**Figure 3**). The significance of all two-way and three-way intersections was estimated using the hypergeometric distribution. Several of these intersections yielded no or few overlapping genes. In particular, given that both LSC (“LSC”, green, solid,) and mobHSC (“Mob”, blue, solid) exhibit substantial expansion of the stem cell population, we were surprised to find no overlap of significantly enriched genes in these populations. This result suggests that self-renewing divisions of mobilized HSC induced in response to Cy/G treatment are driven by pathways distinct from those that drive leukemic expansion upon loss of JunB.

In contrast, intersection of genelists describing targets enriched in MPP relative to HSC, and in non-mobilized and non-leukemic HSC, did yield a short list of 32 clones, representing 30 unique targets. This set represents genes whose expression is increased as HSC differentiate to MPP, but reduced when HSC are mobilized or transformed. Interestingly, this list is highly enriched in nuclear proteins with transcriptional regulatory activity, including Esr1, Fos, Foxp1, Iep5, Laf4, and several zinc finger containing proteins: Zfp148, Zfp36, and Zfp469. A common property of MPP, mobilized HSC and LSC is their enhanced proliferative activity; however, unlike mobilized HSC and LSC, MPP harbor little or no self-renewal activity. Thus, the genes included in this set may represent those whose expression in proliferating cells is incompatible with self-renewal, such that they must be down-regulated to support expansion of self-renewing HSC (mobilization) or LSC (transformation), and are increased in MPP to promote differentiation.

Importantly, intersecting genes selectively enriched in non-mobilized and non-leukemic HSC with genes enriched in HSC when compared to MPP yielded 129 clones representing 93 individual genes (**Figure 3** **and Supplemental Table S5**). The 93 unique genes in this intersection thus are expressed by “normal”, quiescent HSC, and are downregulated during differentiation, mobilization and leukemogenesis. This overlapping dataset is of particular interest as it likely includes genes that are crucial for the steady-state maintenance and regulation of a normal, slowly proliferating HSC.

### Genes Enriched in Normal HSC

**Figure 4:**
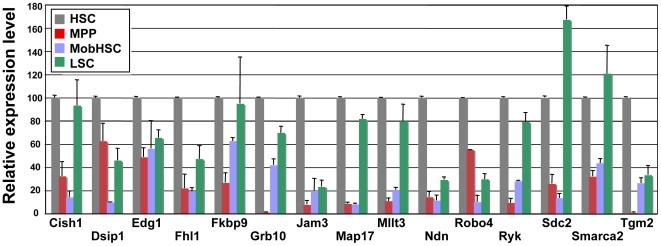
Quantitative RT-PCR of HSC, MPP, D+2 mobilized HSC and LSC. The relative level of expression of genes identified by microarray analysis as downregulated upon HSC differentation, mobilization and leukemic transformation. Normal HSC levels were set to 100. Grey bars, HSC; red, MPP; blue, mobilized HSC; green, LSC.

Using quantitative RT-PCR (qRT-PCR), we validated the differential expression of several of the 93 genes from the 3-way intersect described above using new, independently generated samples (**Figure 4**). All genes in the HSC/MPP and HSC/mobHSC data sets showed the same trend by qRT-PCR as seen in the array analysis. While this concordance of data was also true for the vast majority of genes in the HSC vs. LSC comparison, some genes identified as downregulated by array analysis did not show significant downregulation when analyzed by qRT-PCR in an independent set of sorted samples. This variability likely relates to subtle differences in the precise stage of MPD at which individual *jun*B-deficient animals were sacrificed, reflecting mouse-to-mouse variability in disease progression. Future studies to generate a more refined time course of differential gene expression in *jun*B-deficient HSC may identify the order in which genes are up- and downregulated during leukemogenesis, and their interdependence or importance for disease initiation versus progression.

**Figure 5:**
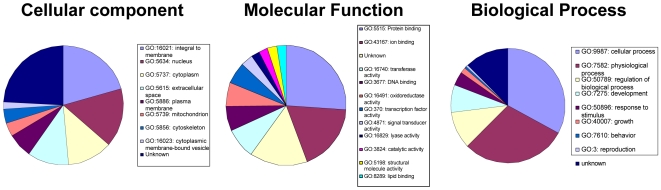
Gene ontology classification of genes selectively expressed by normal, quiescent HSC.

We broadly categorized the 93 HSC “signature” genes based on GO analysis (**Figure 5**) and subcellular location. Enriched expression was observed for several genes encoding extracellular proteins, including biglycan, transglutaminase2, follistatin-like1, angiopoietin1 and procollagen4a2. Expression of several of these in “normal” HSC likely has significant implications for regulation of HSC quiescence. For example, biglycan is an extracellular matrix (ECM) proteoglycan that binds TGF-beta, collagen, and other ECM components. Interestingly, biglycan-deficient mice develop age-associated osteopenia thought to arise from defects in the maintenance and metabolic activity of osteogenic precursor cells [25]. Biglycan deficiency also causes reduced responsiveness to TGFbeta among osteolineage cells [25]. As TGF-beta has been reported as a negative regulator of HSC number, down-regulation of biglycan among differentiating, mobilizing and transformed HSC may insulate them from TGF-beta activity, thus facilitating their enhanced proliferative activity. Similarly, binding of angiopoietin 1 (Angpt1), a secreted cytokine, to its receptor Tie2 on HSC is reported to enhance HSC quiescence and to modulate expression of adhesion molecules implicated in HSC mobilization and retention in the BM [26]. Interestingly, although previous studies have suggested that osteoblasts are the primary producers of Angpt1 in BM, our gene expression data suggest that HSC quiescence can also be modulated by autocrine effects.

Several membrane proteins, including Syndecan2, Edg1, calsyntenin1, Map17, Phemx, Ryk, Itm2a, Jam1 and Jam3, Robo4, Cish, Protein C receptor (PRCR), Prion protein, Mllt4 and Ica1 were enriched in the HSC signature. Several of these receptors are involved in cell migration or location in other systems. Edg1 encodes a receptor for sphingosine 1-phosphate (S1P). S1P recently has been characterized as a unique bimodal regulator of cell migration in the thymus and lymphatic system [27], an important determinant of plasma cell homing from lymph nodes to BM [28], and a key regulator of the physiologic recirculation of HSC and progenitor cells [29]. Thus, downregulation of S1P receptor on HSC may facilitate their egress from the BM niche. Likewise, Jam2 is a leukocyte migration receptor that mediates heterotypic cell-cell interactions with integrin alpha(4)beta(1) (VLA-4), an important receptor in the BM homing and mobilization of HSC [30]. Jam1 and Jam3 were recently shown to be reliable markers for HSC [31], [32]. Thus, the Jam family of adhesion molecules are likely important for HSC retention in the niche. Cish is a membrane protein involved in the downregulation of cytokine responsiveness, potentially acting as a growth inhibitor. Notably, the genomic region encoding Cish is frequently deleted in lung and kidney tumors, implicating this gene in tumor progression and suggesting that its loss in LSC might potentiate leukemic transformation of HSC [33]. Ryk is a tyrosine-kinase-like receptor and a coreceptor for the Wnt signaling protein, previously implicated in the maintenance of mouse HSC [34], [35]. Ryk was shown recently to play roles in neural stem cell differentiation by Wnt-induced translocation to the nucleus [36]. Finally, Robo4 is a homolog of the brain-specific Roundabout receptors that bind the secreted Slit ligands and play roles in axon guidance, while PRCR selectively marks HSC [37] and Prion protein has been implicated in HSC self-renewal [38].

We also observed significant representation in the HSC gene set signature of genes involved in basic metabolic processes, including members of the glycogen phosphorylase (Pygl), aldehyde dehydrogenase (Aldh1a7), guanylate cyclase 1 (Gucy1a3), lactate dehydrogenase (Ldh2), and cytochrome P450 (Cyp2j6) families. Enrichment of these genes may reflect a differential requirement for certain metabolic effectors in the transition from a largely quiescent and metabolically inactive HSC to a highly proliferative and metabolically active cell upon differentiation, mobilization or transformation.

Nuclear proteins enriched among HSC signature genes include cell cycle regulators such as Cdkn1c, Necdin, and Rbbp9, and transcription factors such as Creb3l2, Mllt3, Pbx1, Prdm16, Smarca2 and Zfp467. Creb3l2 belongs to the basic leucine-zipper family of transcription factors and was identified as a balanced translocation fusion partner in low-grade fibromyxoid sarcoma [39]. Ndrg2 is a candidate tumor suppressor gene on chromosome 14q that is inactivated during meningioma progression. Ndrg2 expression is induced by WT1, a transcriptional regulator frequently expressed in leukemias [40]. Mllt3 (AF9) plays roles in hematopoietic differentiation into megakaryocyte and erythroid lineages [41] and as a translocation partner with Mll causes leukemia via a leukemic stem cell mechanism. Thus, Mllt3 plays roles in normal cell growth and maintenance and in oncogenesis [42], [43]. Pbx1 cooperates with Hox proteins and has well-established roles in hematopoiesis [44]–[46]. Intriguingly, it was recently suggested that Pbx1 promotes HSC quiescence and self-renewal [47]. A short form of Prdm16 (MEL1) has been shown to block G-CSF-induced myeloid differentiation [48] and thus may help to maintain the undifferentiated state of HSC, whereas Zfp467 augments and transactivates Stat3 signaling. Nrip1 and Rex3 were proposed to be diagnostic markers of different forms of leukemias [49]. Vilar et. al. suggested that Rex3 links cell surface receptor signaling to the cell cycle and neuronal differentiation by binding the neurotrophin receptor [50]; HSC-selective expression indicates that Rex3 could play similar roles in regulating proliferation and differentiation among hematopoietic precursors. Smarca2, a SWI/SNF-related regulator of chromatin also known as Brm, plays important roles in transcriptional processes via regulation of chromatin modifications and structure. Smarca2 also interacts with TopBP1, a protein involved in DNA replication and the DNA damage checkpoint to promote cell survival [51]. Smarca2 has been linked to inhibition of cell proliferation through interactions with cyclin D3 [52]. In agreement with the differential expression in our data sets, Muchardt et al suggested previously that increased levels of Smarca2 promote the withdrawal of the cell from the cell cycle [53].

Collectively, the genes discussed above define a molecular signature associated with the steady-state quiescent status of HSC, and suggest common targets whose perturbation can lead to enhanced cell proliferation in the context of either differentiation, stem cell expansion, or transformation.

### Comparison to Previous Findings

**Figure 6:**
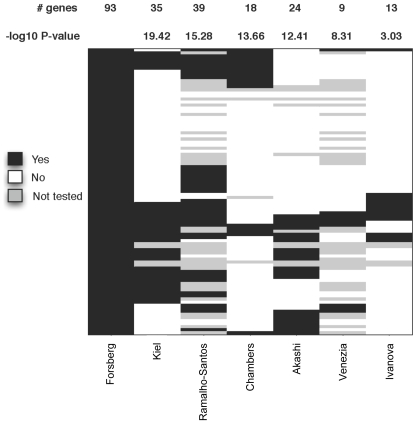
Comparison between the findings in our study and six other reports on HSC gene expression. The 93 genes selectively expressed in normal HSC are indicated in the left column. Additional columns represent the same 93 genes in each study, named below the respective column. Black indicates that the gene was upregulated in HSC; white indicates that the gene was not upregulated; gray indicates that the gene was not tested.

To place the 93 genes in the context of other expression studies in HSC, we compared the similarity between our set and the genes upregulated in six previous HSC studies [54]–[59]. Specifically, we assessed the overlap between the genes identified as upregulated in each of the six studies and our 93 genes and estimated the hypergeometric p-value associated with each comparison. In spite of differences in the identity of the comparison cell type, as well as platforms and assessment of significance of differential regulation, we found a significant overlap between our data set and all other studies (**Figure 6** and **Table 1**). Several genes, notably biglycan (Bgn), protein C receptor (Procr), retinol binding protein (Rbp1), and T-cell specific GTPase (Tgtp) were upregulated in HSC in six out of seven studies.

**Table 1:** Significance of overlap between our study and six other HSC gene expression studies.

Comparison datasets	Genes in common	Max number of genes that could have been identified	Significance of overlap (-log10 p-value)
Kiel	35 (89)	89/93	19.4166
Ramalho-Santos	39 (60)	60/93	15.2799
Chambers	18 (89)	89/93	13.6617
Akashi	24 (83)	83/93	12.4116
Venezia	9 (60)	60/93	8.3054
Ivanova	13 (89)	89/93	3.0312

### Transcriptional Regulators of HSC-Specific Genes

**Figure 7:**
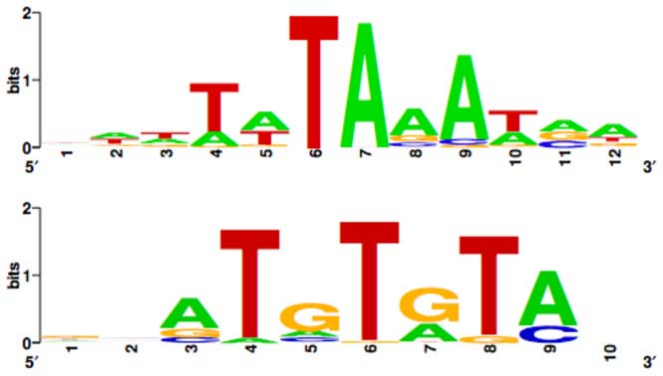
Putative transcription factor binding motifs enriched in upstream regulatory regions of genes selectively expressed by normal, quiescent HSC.

To identify potential master regulators of genes selectively expressed in normal HSC, we performed sequence analysis of the genomic regions upstream of the 93 genes in the common intersect. We searched for both known transcription factor binding sites using the Transfac database (http://www.cbil.upenn.edu/cgi-bin/tess/tess?RQ=NBqt) and for enrichment of novel sequence motifs. The abundance of these sequences in the 3way intersection was compared to both the genome-wide input dataset and to the mouse genome. This analysis identified two sequence motifs enriched with statistical significance in the HSC-selected upstream regions (**Figure 7**). Intriguingly, these sequences may be bound by as of yet unidentified transcriptional regulators, as they do not contain sequence motifs recognized as binding sites for known transcription factors. In addition, we identified binding sites for 467 known transcription factors and sites for 8 of these were significantly enriched in the HSC dataset (**Table 2**). Literature searches revealed that all 8 transcription factors have been implicated in hematopoiesis. In particular, Evi1 is an oncogenic transcription factor in myeloid leukemias, and may regulate normal hematopoiesis by interacting with transcription factors in the Gata family [60], [61]. Deletion of the ubiquitously expressed basic leucine zipper transcription factor AFT4 leads to severe anemia [62]. The interferon response gene IRF1 may act as a tumor suppressor by regulating the differentiation and proliferation of myeloid cells via the Stat pathway [63]–[66]. Nf1 is a tumor suppressor that regulates normal hematopoiesis. Mutations in or deletion of Nf1 leads to myeloproliferative disorders [67], [68]. NF-Y may play roles in the control of HSC self-renewal by inducing the expression of Hoxb4 [69], a known positive regulator of HSC self-renewal [12], [70] as well as other genes involved in HSC self-renewal [71]. NF-Y also has been shown to regulate histone methylation and may function as both an activator and repressor of transcription [72]. Finally, the Ikaros proteins are well characterized in hematopoiesis [73]. Altered ik2, ik4 and ik6 transcripts have recently been identified in Philadelphia chromosome-positive acute lymphoblastic leukemia (Iacobucci 2008), demonstrating that Ikaros and Ikaros target genes play important roles in leukemogenesis.

**Table 2:** Transcription factors with binding sites enriched in the upstream region of genes expressed selectively in HSC.

Evi-1	ecotropic viral integration site 1
ATF4	Activating transcription factor 4
IRF-1	interferon regulatory factor 1
NF-1	neurofibromatosis 1
Ik-2	ikaros 2
c-Myc/Max	cellular myelocytomatosis oncogene
NF-Y	nuclear transcription factor-Y
MAZ	MYC-associated zinc finger protein

It is intriguing to note that the transcription factors we identified in this metaanalysis play important roles in hematopoiesis. It will be of interest to determine the comprehensive set of target genes for these transcription factors in HSC to further build a molecular portrait of the complex networks of genes that regulate normal HSC function.

## Discussion

Gene expression data from highly purified HSC at steady-state compared to non-self-renewing, multipotent progeny, to mobilized HSC, and to leukemic HSC provide an intriguing set of novel target molecules that are commonly induced or repressed as HSC differentiate, expand and mobilize, and undergo leukemic transformation. Each of the pair-wise datasets provide important insights into these specific individual processes at the genome-wide level, and identify new targets to enable specific manipulation of HSC in desired directions. Comparative analysis between datasets identified numerous genes specific for each of the processes, as well as mechanisms or families of downregulated genes common to HSC transition into either of the three fates. In addition to identifying specific molecular regulators and potential targets for manipulation, these datasets also provide insights into how gene expression changes are coordinated at a genome-wide level.

### Increased Understanding of Known and Novel Stem Cell Regulators

As the HSC is one of few stem cells that can be isolated to homogeneity, has proven multipotentiality at the single-cell level *in vivo*, and produces numerous well-characterized and functionally distinct progeny, the hematopoietic system represents a unique opportunity to define the critical molecular regulators of stem cell function and activity. Importantly, the analyses performed in this study predicted a number of molecules known to regulate HSC function. For example, we found that the transcription factor Egr1 was highly significantly downregulated in mobilized HSC (**Supplemental Table S1**). Indeed, it was recently shown that Egr1 regulates HSC migration and location [74], as would be expected from its differential expression described here. Likewise, the adhesion molecule Esam1, identified in the HSC vs MPP analysis, is a highly selective HSC marker [75], [76]. Although it is not surprising that these and multiple other known regulators of HSC function were identified, this concordance of data support the validity of our approaches and suggests that the numerous novel genes discovered here will play important roles in the regulation of HSC function under different circumstances. Interestingly, a significant proportion of the differentially expressed genes encode unknown genes or uncharacterized EST sequences, illustrating the fact that a substantial portion of the transcriptome of HSC remains unexplored. Indeed, GO analysis indicates that the largest category of transcripts selective for normal HSC fall in the “unknown” categories in cellular location and molecular function (**Figure 5**). Thus, this report opens new avenues of investigation at the molecular level that will contribute to a more complete understanding of HSC function.

By performing meta-analysis of differentially expressed gene sets we defined a unique expression profile highly selective for quiescent HSC. These “HSC signature genes” likely include active regulators of controlled self-renewing divisions and interactions with the microenvironment that function to actively suppress differentiation, proliferation, and transformation. In support of this premise, many genes selective for normal HSC have been shown to function in growth suppression and to prevent leukemic transformation. As in the individual pair-wise comparisons, this analysis revealed a combination of novel genes and molecules with known roles in HSC function. Further characterization of known and novel genes will be critical to enhance strategies for preventing and controlling leukemias, as well as optimizing conditions for HSC expansion and mobilization.

### Transcriptional Control of HSC Function

It is intriguing to note that all of the transcription factors identified by the DNA sequence analysis as enriched among HSC signature genes have known roles in hematopoiesis. This fact underscores the value of this analysis and suggests that identifying the factors binding to the novel DNA binding motifs described in **Figure 7** will be fruitful for understanding transcriptional regulation in HSC. In addition, the demonstration that binding sites for known transcriptional regulators are enriched in normal HSC will shed light on the mechanisms of action of these factors. Further analysis may indeed uncover how these factors converge on critical target genes and explain functional redundancy or synergism. Recent technical advances also makes it possible to assess enzymatic chromatin modifications at sites of interest [77]. Combinatorial control to amplify or counteract transcriptional signals likely balances critical decisions such as proliferation versus quiescence or self-renewal versus differentiation. Pursuing the mechanisms of master regulators promises to be a rewarding endeavor for understanding the complexities of HSC function.

### Self-Renewal vs. Proliferation in the Control of Stem Cell Activity

While self-renewal capacity is attenuated as HSC differentiate into MPP, self-renewing divisions increase upon mobilization and in leukemic HSC. The existence of selective groups of up- and downregulated genes in these scenarios supports the supposition that distinct molecular pathways function to signal self-renewal in different contexts. This finding is consistent with our original hypothesis that “induced” HSC self-renewal, as in the context of hematological injury mimicked by mobilization or in response to oncogenic signals, engages an at least partially distinct group of regulatory and signaling proteins as compared to HSC self-renewal under homeostatic conditions. These molecular signatures are defined by the genes and pathways described above. The data of this report brings us closer to the complete understanding of co- and antiregulation of functional entities necessary to fully appreciate the function of HSC in steady-state and regenerative hematopoiesis, and to pinpoint targets of oncogenic transformation.

## Materials and Methods

### Mice

All experiments were performed using young adult (8–12 weeks old) C57BL/Ka-Thy1.1 or JunB-deficient mice on C57Bl6 background. Cy/G treatment was performed as in (Passegue 2005). Mice null for JunB were described previously [22]. All mice were maintained in Stanford University's Research Animal Facility in accordance with Stanford University guidelines.

### Flow Cytometry

Antibody staining and cell sorting were performed as previously described [3], [5], [24]. Hematopoietic cell populations were derived from bone marrow isolated from murine femurs and tibias, unless otherwise noted. Surface marker phenotypes are provided in **Table 3**. Typically, cells were isolated by incubation with unconjugated lineage antibodies (CD3, CD4, CD5, CD8, B220, Gr1, Mac1, and Ter119), followed by goat anti-rat Tricolor (Cy5PE; Invitrogen), and c-kit-APC, Sca1-TxR, Flk2-PE (eBioscience) and Thy1.1-FITC. Antibodies were purified and conjugated by standard procedures in the Weissman laboratory, unless purchased commercially as stated above. Functional assessment of these populations have been performed in multiple studies [3]–[6], [8], [9], [11], [17], [18], [74], [76], [78]–[81].

**Table 3:** Immunophenotyping strategies used to identify stem cells in this study.

Population	Immunophenotype	References
HSC	ckit^+^Thy1.1^lo^Lineage marker^−^Sca-1^+^Flk2^−^ (KTLSF^−^)	Cao, 2004; Christensen, 2001; Forsberg, 2006; Forsberg, 2005; Min, 2008; Ooi, 2009; Passegue, 2005
MPP	ckit^+^Thy1.1^−^Lineage marker^−^Sca-1^+^Flk2^+^(KT^−^LSF^+^)	Cao, 2004; Christensen, 2001; Forsberg, 2006; Forsberg, 2005; Min, 2008; Ooi, 2009; Passegue, 2005
MobHSC/nonMob	ckit^+^Thy1.1^lo^Lineage marker^−^Sca-1^+^ (KTLS)	Morrison, 1997a; Morrison, 1997b; Morrison, 1994; Wright, 2001; Passegue, 2005
LSC/nonLSC	ckit^+^Lineage marker^−^Sca-1^+^Flk2^−^ (KLSF)	Passegue, 2004; Santaguida, 2009

### cDNA Array Hybridization

Sample processing was performed as described previously [24]. Briefly, RNA was isolated and amplified from HSC double-sorted by FACS from the BM of pools of untreated or day +2 Cy/G-treated C57BL/6-Ka/Thy1.1 mice (50,000–100,000 cells); or from the BM of individual control or *jun*B-deficient mice diagnosed with a pre-leukemic MPD (6,000–13,000 cells). RNA was amplified, dye-labeled with Cy3 or Cy5 and hybridized to custom spotted cDNA arrays [24] made available through the Stanford Microarray Core Facility (Stanford University) and containing 42,025 cDNAs and expressed sequence tags (ESTs) from a wide variety of tissue- and developmental stage-specific libraries, including a library made from BM-subtracted HSC cDNA [82] and a selection of hand-picked genes involved in development and hematopoiesis [24]. To obtain accurate and reliable transcription profiles, we performed pairwise competitive hybridizations in three independent experiments, each with a “dye swap”, so that each comparison was performed 6 times. Slides were scanned and gridded, and the resulting data files were submitted to the Stanford Microarray Database (http://genome-www.stanford.edu/microarray) to assign identity to each spot and to normalize Cy3/Cy5 relative intensities for each slide. To identify differentially expressed genes, we employed stringent criteria of reproducibility, as in [24], requiring a particular spot to be present in at least 5 out of the 6 pairwise comparisons. Features passing this criterion were analyzed for statistically significant differences using Significance Analysis of Microarrays (SAM, http://www-stat.stanford.edu/~tibs/SAM/) [23]. Significantly differentially regulated genes were defined by SAM at a false discovery rate of 10%. Microarray data reported in this manuscript is described in accordance with MIAME guidelines.

### Quantitative RT-PCR

qRT-PCR was performed as described previously [5]. Primer sequences can be obtained upon request.

### Identifying Genes Corresponding to Probes on the Microarrays

For each of the 40,681 unique clones, we obtained the nucleotide sequence in NCBI's mouse EST sequence database downloaded from ftp://ftp.ncbi.nih.gov/genbank/gbest. We identified the predicted gene target for each clone by searching for the best-matching transcript matching the clone. We used BLAST [83] to compare each clone's nucleotide sequence to all transcripts contained in release 7 of the mouse KnownGenes track from the UCSC Genome Browser's database. We associated each clone with the NCBI EntrezGene identifier of the best-matching gene. We found EST sequences for 34,838 (86%) of the clones on the microarrays, of which we could identify best-matching genes for 32,402 (93%). These clones mapped to 12,903 unique EntrezGene identifiers. Thus, each gene was represented by 2.5 clones on average.

### Identifying Significant 3-Way Overlaps

A series of microarray analyses produces a set of clone lists, *{C_1_, C_2_, … C_L_}*, where each *C_i_* contains a list of clone identifiers corresponding to the probes found to be significantly up- or down-regulated under condition *i*. Each clone identifier in *C_i_* was mapped to its corresponding EntrezGene identifier as described above, and a non-redundant set of genes *G_i_* was produced by removing any duplicate EntrezGene identifiers. We searched for statistically significant overlaps between any two combinations of gene lists *G_i_* and *G_j_*. We calculated a binary overlap score *B_ij_*, such that the probability of an observed overlap or better would occur by chance is given by the hypergeometric distribution:
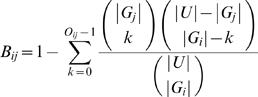
where *|A|* is the number of genes in list *A*, *O_ij_* is the number of overlapping EntrezGene identifiers in *G_i_* and *G_j_*, i.e. (*|G_i_∩G_j_|*), and *U* is the set of all genes with detectable levels of expression on the microarrays used in our study. A gene was considered detectable if it had one or more clones that were detectable. A clone was considered detectable if it had expression levels above background in at least five out of six replicate hybridizations in at least one of the microarray data sets.

We calculated binary overlap scores between all pairwise gene lists. We then search for significant three-way overlaps which are defined as a set of three gene lists, *G_i_, G_j_, G_k_* such that all three binary overlap scores, *B_ij_*, *B_ik_*, *B_jk_* are significant. More formally, we compute a trinary overlap indicator *T* *_ij_*, based on all two-way intersections with the corresponding original gene lists:
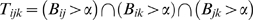
where *α* is the significance level (in this study we used *α* = 0.001). *T* *_ijk_* evaluates to true if all pairs of overlaps are significant at the *α* level.

### Comparison to Previous Gene Expression Studies

For each study in **Figure 6** and **Table 1**, we mapped the probes identified as upregulated in the hematopoietic stem cell population to Entrez Gene. In order to perform a fair comparison between the studies, we restricted the evaluation to only the genes that were commonly tested between each specific pair of studies. This step reduced the total number of genes that could have been identified as significant from the original upregulated sets. The p-value was assessed based on the hypergeometric distribution and is presented in –log10 space (**Table 1**).

## Supporting Information

Table S1 Array features downregulated in day +2 mobilized HSC compared to HSC at steady-state.
(0.38 MB XLS)

Table S2 Array features upregulated in day +2 mobilized HSC compared to HSC at steady-state.
(0.04 MB XLS)

Table S3 Array features downregulated in HSC from the BM of JunB-deficient mice (LSC) compared to HSC from wt mice.
(0.19 MB XLS)

Table S4 Array features upregulated in HSC from the BM of JunB-deficient mice (LSC) compared to HSC from wt mice.
(0.02 MB XLS)

Table S5 Genes enriched in normal, steady-state BM HSC from wt mice compared to MPP, LSC, and mobilized HSC.
(0.05 MB XLS)
